# Supplementary material for: FTIR Spectroscopy as a Tool to Study Age-Related Changes in Cardiac and Skeletal Muscle of Female C57BL/6J Mice
Source: Molecules. 2021 Oct 23;26(21):6410. doi: 10.3390/molecules26216410 (PMC8587752; doi:10.3390/molecules26216410)
Supplement: Supplementary file 1 [file molecules-26-06410-s001.zip › molecules-1400691-supplementary.pdf]

## SUPPLEMENTARY DATA

## FTIR SPECTROSCOPY AS A TOOL TO STUDY AGE-RELATED CHANGES IN CARDIAC AND SKELETAL C57BL/6J FEMALE MICE MUSCLE

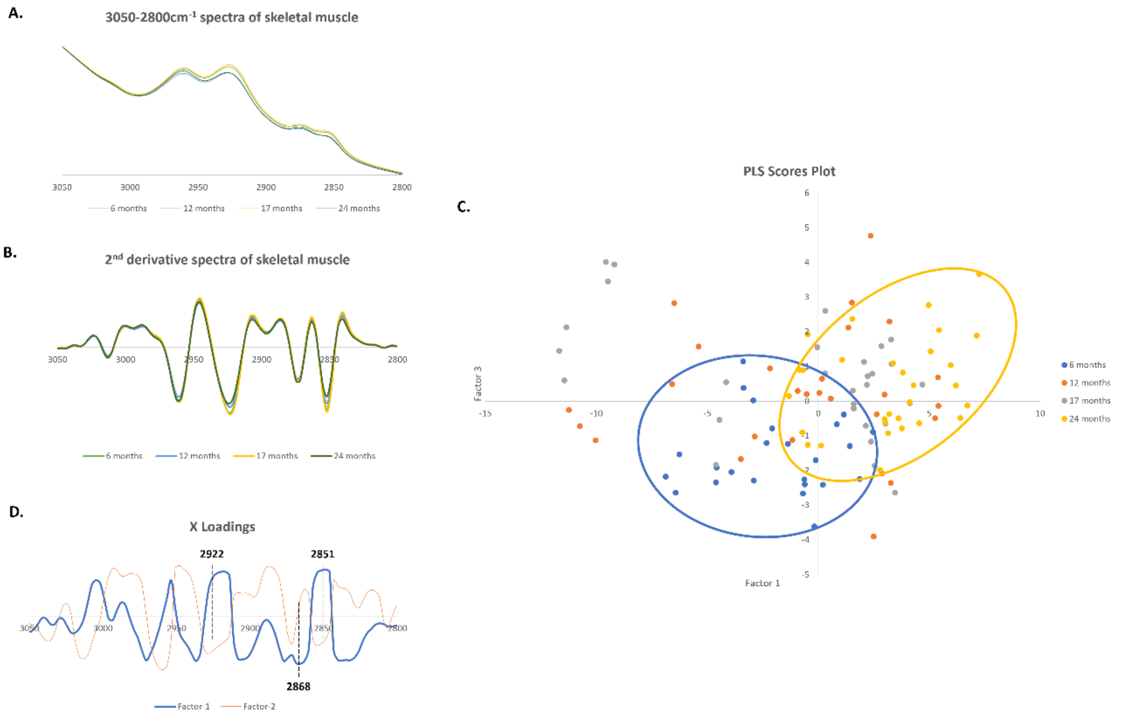


**Figure S1:** PLS analysis of skeletal muscle in the 3050-2800cm$^{-1}$ spectral region. **A**. Normalized spectra of skeletal muscle in the 3050-2800cm$^{-1}$ region. **B.** Second derivative spectra, used for PLS model; **C.** PLS scores plot, showing a discrimination by Factor 1 of younger samples (blue circle) from older samples (yellow circle). **D.** X loadings responsible for discrimination of samples, with main peaks highlighted in bold.

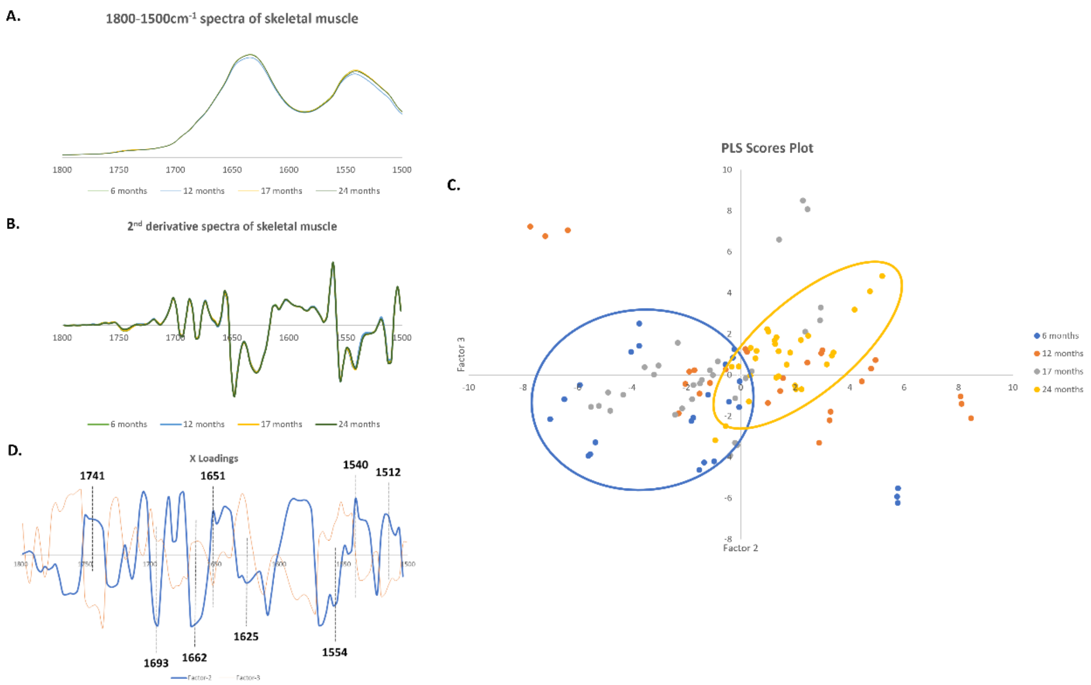


**Figure S2:** PLS analysis of skeletal muscle in the 1800-1500cm$^{-1}$ spectral region. **A.** Normalized spectra of skeletal muscle in the 1800-1500cm$^{-1}$ region. **B.** Second derivative spectra, used for PLS model; **C.** PLS scores plot, showing a discrimination by Factor 2 of younger samples (blue circle) from older samples (yellow circle). **D.** X loadings responsible for discrimination of samples, with main peaks highlighted in bold.

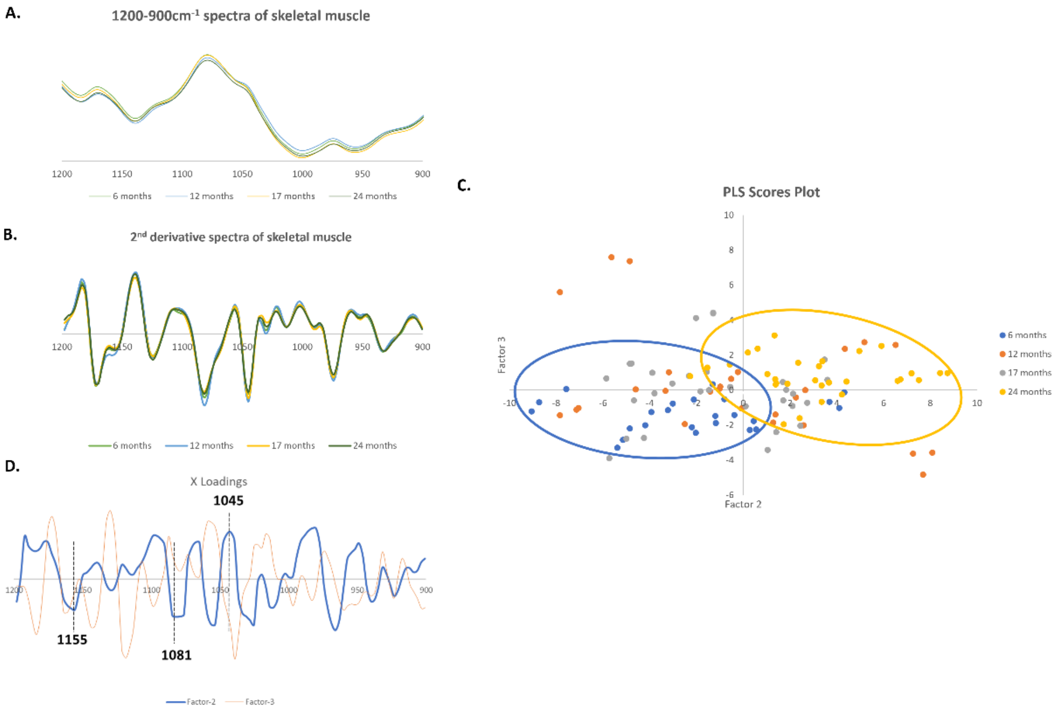


**Figure S3:** PLS analysis of skeletal muscle in the 1200-900cm$^{-1}$ spectral region. **A.** Normalized spectra of skeletal muscle in the 1200-900cm$^{-1}$ region. **B.** Second derivative spectra, used for PLS model; **C.** PLS scores plot, showing a discrimination by Factor 2 of younger samples (blue circle) from older samples (yellow circle). **D.** X loadings responsible for discrimination of samples, with main peaks highlighted in bold.

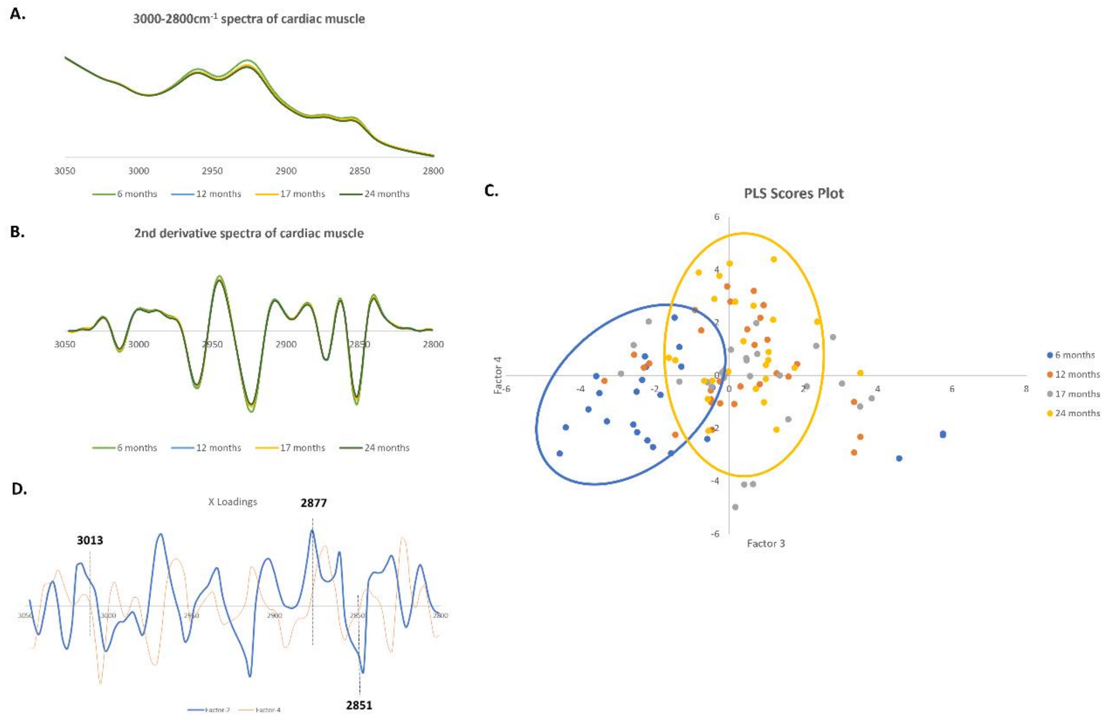


**Figure S4:** PLS analysis of cardiac muscle in the 3050-2800cm$^{-1}$ spectral region. **A.** Normalized spectra of cardiac muscle in the 3050-2800cm$^{-1}$ region. **B.** Second derivative spectra, used for PLS model; **C.** PLS scores plot, showing a discrimination by Factor 3 of younger samples (blue circle) from older samples (yellow circle). **D.** X loadings responsible for discrimination of samples, with main peaks highlighted in bold.

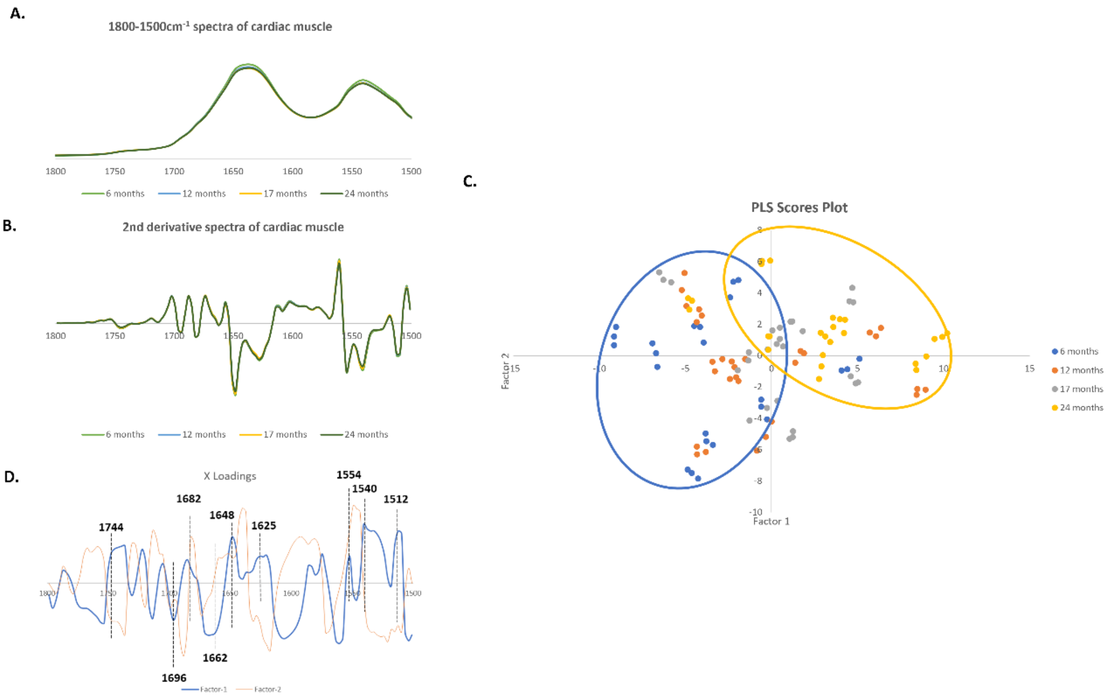


**Figure S5:** PLS analysis of cardiac muscle in the 1800-1500cm$^{-1}$ spectral region. **A**. Normalized spectra of cardiac muscle in the 1800-1500cm$^{-1}$ region. **B.** Second derivative spectra, used for PLS model; **C.** PLS scores plot, showing a discrimination by Factor 2 of younger samples (blue circle) from older samples (yellow circle). **D**. X loadings responsible for discrimination of samples, with main peaks highlighted in bold.

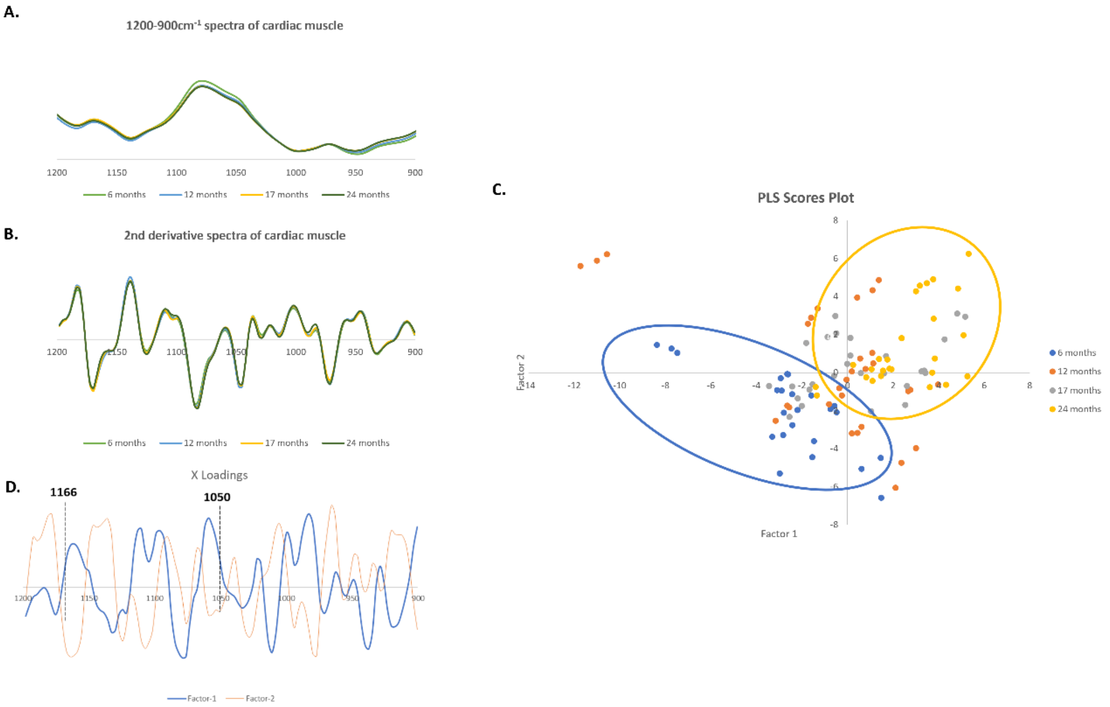


**Figure S6:** PLS analysis of cardiac muscle in the 1200-900cm$^{-1}$ spectral region. **A.** Normalized spectra of cardiac muscle in the 1200-900cm$^{-1}$ region. **B.** Second derivative spectra, used for PLS model; **C.** PLS scores plot, showing a discrimination by Factor 1 of younger samples (blue circle) from older samples (yellow circle). **D.** X loadings responsible for discrimination of samples, with main peaks highlighted in bold.

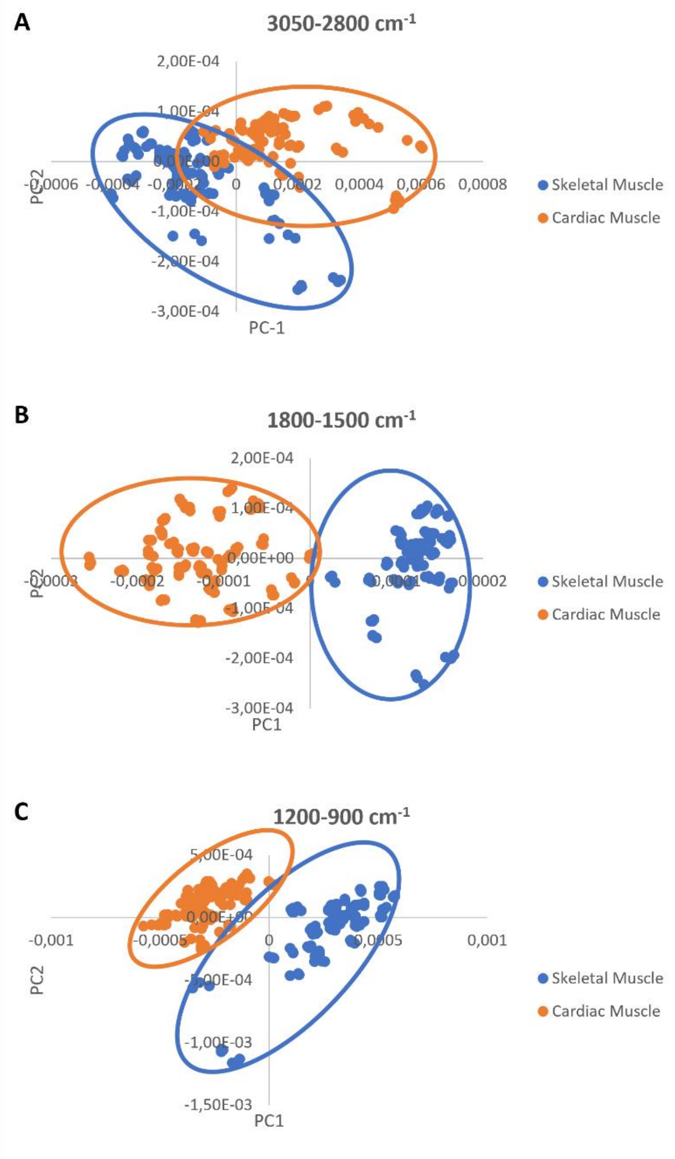


**Figure S7:** PCA exploratory analysis of FTIR spectra of skeletal and cardiac muscle in the **A.** 3050-2800cm-1, **B.** 1800-1500cm-1 and **C.** 1200-900cm-1 spectral regions.
